# An Analysis of Trauma and Elective Orthopaedic Incomes in a UK Hospital: A Rationale for Implementation of Ambulatory Trauma

**DOI:** 10.7759/cureus.101527

**Published:** 2026-01-14

**Authors:** Prateek A Saxena, Neil Ashwood, Niyam Amanullah, Paul Wilson

**Affiliations:** 1 Orthopaedic Surgery, University Hospitals of Derby and Burton National Health Service Foundation Trust, Burton, GBR; 2 Trauma and Orthopaedics, University Hospitals of Derby and Burton National Health Service Foundation Trust, Derby, GBR; 3 School of Engineering, Computing and Mathematical Sciences, University of Wolverhampton, Wolverhampton, GBR

**Keywords:** acute trauma care, ambulatory surgery centers, ambulatory treatment, economic burden of healthcare, financial impact, medical coding, net income

## Abstract

Introduction

In the UK, one-third of orthopaedic departments experience financial losses from surgical interventions, with specialist hospitals experiencing higher deficits due to factors like patient age and injury severity. This study investigates financial modelling and potential changes in care delivery to enhance the regional flow of orthopaedic care throughout the UK, as well as strategies to address this economic disparity.

Methods

The study analyzed the impact of various orthopaedic subspecialties on departmental income and loss during the previous financial year (April 2023 to April 2024). The goal was to understand how different care models, including both ambulatory (same-day) and non-ambulatory (inpatient) pathways, influence these financial outcomes. The need for non-ambulatory care increases with patient frailty and age; however, the study suggests that treating fitter trauma patients on an outpatient basis could help offset overall orthopaedic care costs.

Results

In the last financial year, the trust encountered 32088 coded episodes, with 1707 trauma and 1275 elective operations. Despite incentivised weekend lists to reduce waiting lists, the demand for trauma care outweighs that for elective care at a local level.

Discussion

The government has significantly invested in elective centres designed to perform high-volume, low-risk operations. These initiatives aim to shorten waiting lists and tackle the backlog of orthopaedic care patients that grew rapidly during the COVID-19 pandemic. Organisational care delivery is largely shaped by economic drivers mandated through government policy, rather than being primarily guided by clinical needs. Trauma patients frequently struggle to receive urgent care within specified time frames due to insufficient funding, unlike elective procedures, which face politically driven, rather than clinical, time limits.

Conclusions

Implementing ambulatory trauma care can help reduce costs and enhance hospital profitability. This approach ensures that patient flows align with demand and provides a payment structure that supports younger, working-age patients returning to employment, provided the day-case surgery tariff is sufficient to support the development of regional ambulatory trauma units.

## Introduction

In 2005, the National Health Service introduced Payment by Results to provide a pricing framework (Tariff) that accommodated geographical demand and service variability [1]. Around the same period, the 2004 NHS improvement plan set an 18-week target for referral to treatment for elective orthopaedics, which has been influenced by the COVID-19 pandemic’s heightened demand for services. Orthopaedics remains the fourth busiest specialty, with waiting lists increasing nearly two-thirds annually, leading to extended wait times each year. Costs and pathways have not been communicated to surgeons or patients until recently, to deal with this demand effectively. In 2002, elective hubs were introduced to boost productivity, reduce cancellations, enhance staff and patient experience, ensure financial sustainability, and enhance care quality [2]. NHS England’s Get it Right First Time (GIRFT) program introduced the high-volume low-complexity (HVLC) programme in May 2021, supporting 108 surgical hubs across the country as of September 2024 to adopt best clinical and operational practices [3]. This allowed for simple cost-effective elective cases to be siphoned off to dedicated units, leaving complex cases with more costs at the local hospitals, where services are also stretched trying to manage trauma cases and deal with issues related to winter care pressures [4].

However, a third of surgical cases in orthopaedics relate to fracture care, which requires timely surgery as outlined by the Orthopaedic Trauma Society [5]. In 2016, it was reported that the incidence of fractures in those over 50 years was 116.3 per 10,000 people [6]. Globally in 2019, fractures accounted for 25.8 million years lived with disability (YLDs), and this had increased by 65.3% since 1990 [7]. The ageing population presents primarily as non-ambulatory trauma cases with fragility fractures, where funding follows a best practice model [8]. Ambulatory trauma can be managed more cost-effectively through day case pathways [9]. However, now that block contracts are in place, it's unclear exactly which cases generate income. The study aimed to review the income and costs for procedures undertaken within the trust over the last financial year to identify potential cases it might import from another trust within the region.

The system uses healthcare resource group (HRG) codes to group treatments into similar cost brackets that have the same code. A price or tariff is derived from each hospital patient episode affected by the patient’s comorbidities, age, and length of stay [1]. The patient’s registered Primary Care Trust (PCT) is then billed accordingly, with each health resource group setting the payment level dependent partly on the national average cost for all hospitals, with additional payments for excess bed days past a 'trim point' that varies depending upon geographical location and local negotiations of the payment [10].

The process of coding is complex, and coders are trained to classify the surgical procedure performed using a classification first published in 1970 by the Office of Population Censuses and Surveys [11]. This has been revised several times, with the fourth version being launched in the 1990s, with different iterations being currently available [11]. The alignment of this with the rapid advancement in procedure scope and technical complexity, such as the introduction of robotic surgery, is highly questionable. In the same way, the patient's diagnosis and co-morbidities are coded using the International Classification of Diseases version 11 [12]. Each HRG code represents a tariff, which is the average cost of a treatment nationwide, though minor regional adjustments are made to reflect the cost of living.

Hospitals calculate costs by applying a standard set of rules set out in the NHS costing manual. The accuracy of the costs is audited and validated to a set of stringent rules, and in one paper by Longo et al., it averaged £2987 for non-specialist orthopaedic units within the UK [10]. Costs are influenced by several factors, including the admission types, like non-elective (short or long), elective, and day case. The rules that are applied aim to capture the full cost of the services delivered, secondly, allocate costs through 'direct imputation' rather than through apportionment, and finally, the costs match the services generating them [10]. The costing process groups costs into those that are directly attributable to patients (e.g., doctors, nurses, drugs). Then costs that are linked to patients to sustain them each day (e.g., linen, catering) and overhead costs that are not related to patients (e.g., senior managers, administrative employees). Such costs are then attributed to different treatment and support services (e.g., pharmacy, building maintenance), to hospital specialties (e.g., general surgery, orthopaedics), to wards, and finally to HRGs [10].

The primary aim of this study was to conduct a comprehensive analysis of the income and expenditures related to orthopaedic trauma and elective surgical procedures within a district general hospital in the United Kingdom during the 2023-2024 fiscal year, with the intention of evaluating the financial viability of transitioning suitable cases to ambulatory (day-case) care pathways and recognizing potential avenues for regional service enhancement.

## Materials and methods

A financial service evaluation was conducted at a patient level within a hospital trust, using data from April 2023 to April 2024, to determine how the care journey is financially supported. The district general hospital, which has 460 beds, serves a population of 360,000 for both elective and level three trauma cases, such as fragility fractures and ambulatory trauma. One-third of cases are appropriate for day surgery, which helps reduce bed occupancy and manage winter bed pressures. 

Trauma cases are tracked via a local registry, while elective arthroplasty patients are identified through the joint registry and electronic patient records. The authors analysed a sample of cases from all subspecialties (hip, knee, foot and ankle, hand, elbow, and shoulder) to understand how the financial performance supports the move to ambulatory care. The findings may help inform the regional flow of patients to level 3 units, assisting busier departments in managing their trauma workflows.

## Results

Based on the financial data for the year, the trust recorded 32,088 orthopaedic episodes charged for at the site. The total number of episodes delivered across outpatient services, virtual appointments, and surgical theatres was slightly higher at 32,363.

The total cost associated with this activity amounted to £26,549,002. However, the income received by the trust for these services was only £21,979,867, resulting in an overall economic loss of £4,569,135 for the year, as detailed in the summary provided in Table 1.

**Table 1:** Activity as it relates to financial performance between April 2023 and March 2024 within Orthopaedics within the Trust. N: Number of cases

Activity	N	Cost	Income
Clinic	22253	£3,278,132	£2,403,657
Virtual	5568	£621,558	£1,037,352
Non-elective	1707	£13,007,172	£8,485,396
Elective	1275	£7,966,305	£8,514,599
Day Case	905	£1,669,493	£1,534,983

In the contract preparations, the planned activity was for 28,191 face-to-face clinic visits, a target that was successfully met with 28,196 completed episodes [1]. Notably, 5,568 of these were virtual appointments, which generated a profit of £415,794 at a cost to the trust of £621,558 [1].

The attributable costs for these virtual appointments appeared particularly high because each case involved a phone call to the patient, a computer clinic, further investigations (MRI or CT scans), and the involvement of one nurse, one surgeon, and administrative support [1]. In total, six people participated in this specific care process, a significantly smaller team compared to the 24 to 30 people directly involved in delivering an inpatient operation or the 10 to 12 people required for a day case operation (excluding administrative support) [1].

The total financial loss across 1,707 trauma cases was £4,521,776 (Table 2). While this is understandable for lengthy stays associated with conditions like femoral fragility fractures (where patients may shift from surgical to medical care models), the high loss per case for hip and fragility fractures is puzzling, given these cases already have the highest morbidity and mortality rates [13]. 

**Table 2:** Income and loss for trauma cases. N: Number of cases

Subspeciality	N	Number of Codes	Average Income	Average Cost	Loss per Case
Foot	99	7	£2320	£4690	£2370
Ankle	181	6	£3487	£7104	£3617
Shoulder	120	3	£6668	£8799	£2131
Clavicle	19	5	£4039	£4339	£300
Elbow and Forearm	186	5	£5117	£7434	£2316
Hand	180	13	£2566	£2805	£239
Wrist	138	8	£3429	£4030	£601
Hip and Femur	577	11	£8577	£14947	£6370
Knee and Tibia	232	8	£5437	£8539	£3102

The significant variation in trauma care costs contributes to these losses. Complex shoulder and elbow reconstructions, for example, generate several thousand pounds of loss per case due to the need for loan instrument kits and implants [13]. Foot and ankle trauma cases, which are becoming increasingly complex with evolving treatment methods and often require staged care, are the next most expensive area, followed closely by the knee [13]. The British Elbow and Shoulder Society has previously raised concerns about inadequate remuneration for elective tariffs, leading to efforts to renegotiate prices [13]. The department's move toward ambulatory care, accelerated during the COVID-19 pandemic, has been helped by allowing care to be delivered in a semi-elective fashion, a model also adopted for upper limb care within the region [13].

Table 3 demonstrates elective care areas that have a favourable income with the tariff process for elective knee, hip, and shoulder arthroplasty helping to balance the books. The move to shorter lengths of stay within the trust and nationally has helped to deliver this effective care. However, anything that increases the theatre time, such as robotic surgery, may reduce the profitability of these procedures within the trust.

**Table 3:** Income and loss for elective cases. N: Number of cases

Subspeciality	N	Number of codes	Average Income	Average Cost	Profit	Loss
Foot and Ankle	146	8	£1746	£2980		£1134
Shoulder	84	4	£7518	£7165	£363	
Elbow	103	7	£2907	£2964		£57
Hand	233	14	£3345	£4357		£1012
Hip	572	4	£7260	£5359	£1901	
Knee	732	2	£7409	£6031	£1378	

Impact of co-morbidities

In the elective hip arthroplasty, based on the Charlson comorbidity index (CCI), the income and costs associated with each case are demonstrated in Table 4. A £231 increase in income was offset by £252 increase in costs, so there was a minimal impact; this was similar for other subspecialties.

**Table 4:** Income and costs per case in elective hip arthroplasty based on the Charlson Comorbidity Index.

Charlson Comorbidity Index	Income	Cost/expenditure
0-1	£7020	£6601
2-3	£7251	£6853

Within this, the costs continued to vary, and while the length of stay had an impact on this, other factors such as implant cost, hired equipment costs, and theatre time appeared to have a greater influence on the remaining cost variability (£618 to £2647) when it came to wrist trauma. All of the operations were undertaken in the same location and had the same Charlson index.

Impact of length of stay

Tables 5, 6 demonstrate profit/loss based on the impact of length of stay for elective hip arthroplasty patients. The length of stay in trauma cases ranged from 2 to 83 days, with a median of 19 days, and this increased the financial strain. Delaying theatre time impacts the balance of lists and increases the departmental expenditure.

**Table 5:** Income and costs for patients undergoing elective hip arthroplasty with CCI of 0-1. CCI: Charlson comorbidity index

No of days	Cost (£)	Income (£)	Profit (£)
1	5530	7829	2299
2	5685	6303	618
3	6688	8315	1627

**Table 6:** Income and costs for patients undergoing elective hip arthroplasty with CCI of 2-3. CCI: Charlson comorbidity index

No of days	Cost (£)	Income (£)	Profit (£)
1	5440	6874	1434
2	5695	6584	889
3	6359	9006	2647

Impact of theatre time on surgical costs for wrist fractures

Surgical procedures exhibit a wide range of theatre costs and durations: the most challenging cases averaged 95 minutes and cost £6,239, while less complex cases averaged 32 minutes at £2,292, with an overall average cost of £69 per minute (£66-£72). Current standard tariffs do not fully account for this variance, only stratifying cases by broad categories (minor, intermediate, major, and very major), rather than specific surgical complexity. This makes accurately predicting surgical time based on the case's complexity and the surgical team's experience level difficult. Furthermore, the time spent training surgical teams creates an additional cost burden.

Delivering wrist fracture care for children and adults who have significant comorbidities and are unsuitable for day care units requires main-site operating rooms to balance surgical lists. This approach increases costs; 13 operations performed on adults in this manner over the year resulted in an average loss per case of £1,474. Of those, five cases were profitable (totalling £3,530, for an average of £706 per case), but eight resulted in a total loss of £22,689 (an average of £2,836 per case). Since the COVID-19 pandemic, more cases are performed within the ambulatory care setting, reducing the average loss to £318 per case for 41 cases delivered within the day case setting. Within that group, 24 cases made a loss (£23,833 total, an average of £993 per case), while 17 made a profit (£10,812 total, an average of £636 per case). Theatre time for these patients charged at £66 per minute rather than the national figure of £38; consequently, reducing overheads would make this shift more favourable. When financial coders were questioned about these excessive costs, they had a challenging time figuring out the discrepancy.

A financial model has been developed to support ambulatory trauma care, which has successfully reduced financial loss within a single trust. However, this model has not yet been implemented on a regional basis. The transition to a day surgery model for trauma shoulder cases has had a positive financial impact. A study of 13 day-case trauma shoulder operations revealed an average profit of £551 per case, with an average cost of £5,966 and an average income of £6,517 over an average stay of just 0.72 days. In contrast, 13 patients receiving elective care for similar issues experienced an average loss of £708 per case, with a longer average stay of 2.18 days (average cost: £7,841; average income: £7,133). Notably, for six cases where trauma shoulder surgery required inpatient stays, often for unavoidable social reasons, the financial outcome was a significant average loss of £1,131 per case. These inpatients had an average stay of 5.42 days, incurring an average cost of £8,799 against an average income of £7,668.

## Discussion

In the last financial year, the trust encountered 32,088 coded episodes, including 1,707 trauma and 1,275 elective operations. Despite incentivized weekend lists aimed at reducing waiting lists, the local demand for trauma care outweighs that for elective care. The National Institutes of Health (NIH) has stated that funding for trauma is not commensurate with its burden of disease, a finding borne out in most countries [14]. 

A prospective study of financial data in a district general hospital conducted in 2011 over a one-week period used health resource group coding to identify 48 trauma patients, who generated £171,941, and 71 elective patients, where the income generated was £150,318 for a typical week within the unit [1].

The current problem is that focusing solely on bed occupancy, rather than other associated costs, has led to an underestimation of elective costs, which average out at close to £3,000 per patient [10]. Using the average cost per patient per episode within the UK, the cost for delivering care to the 71 elective cases may approach £212,000 a week [1,10]. A third of trusts do not make financial gains through orthopaedic surgery, and understanding these costs and how to mitigate them may help improve the resources available to treat more patients.

Payment by results (PbR) was introduced to reward hospitals for productivity. In orthopaedic clinics, there is a separate tariff for two specific procedures: removal of sutures (ROS) and attention to dressing [15]. However, the inability of hospitals to accurately document and code the activity involved in orthopaedic care, including clinic operations, often leads to lost income from failed claims [15]. 

The Office of Censuses & Surveys (OPCS) codes used for determining this income have not had a major revision since the 1990s, despite four versions being produced between 1950 and 1990. The fourth revision, OPCS-4 (Office of Population Censuses and Surveys Classification of Interventions and Procedures, Fourth Edition), published in 1990, had 1,183 categories that were subdivided, resulting in over 4,000 valid codes. This means the current codes significantly lag modern techniques and the cost of delivering those techniques, such as arthroscopic shoulder surgery.

The purpose of OPCS-4 is "to permit the systematic recording, analysis, interpretation, and comparison of surgical procedure and intervention data collected in the NHS," and this version is on update 11.1. The fourth revision of OPCS had objectives that classified current surgical operations, with reference to the incorporation of recent innovative techniques [11]. While it aimed to eliminate rarely performed operations, it included flexibility to code procedures not requiring the full operating theatre environment, was to be responsive to less defined specialty boundaries, and was capable of future expansion, being completed by 1995 [11]. OPCS-4.3 was the next major iteration to support the Department of Health Financial Flows project, known as PbR, which started in 2002 and was completed four years later. Since 2007, stakeholders have been able to request changes to the codes. 

It remains a complex process where medical terminology is translated into a set of codes, capturing all the codes relevant to that episode in the correct sequence. Health care providers who have implemented an Electronic Patient Record (EPR) system use clinical terminology outlined by the Systematised Nomenclature of Medicine Clinical Terms (SNOMED CT 2024), and these computer systems can use the national maps between the SNOMED CT UK Edition and OPCS-4 to help create the correct codes (SNOMED CT 2024). SNOMED CT is used by General Practitioners (GPs) and is recommended by the National Information Board (NIB) to bring clarity and uniformity to clinical terminology.

However, have the codes kept up with the rapid progression in surgical techniques and costs? For instance, shoulder arthroscopy was only introduced for the first time with a therapeutic manoeuvre of subacromial decompression in 1985 by Harvard Ellman. Up until then, this procedure had been diagnostic only, having been first described in cadavers in 1931 and subsequently in patients in 1965. Now, complex procedures are performed, such as the Latarjet procedure, first performed by Lafosse in 2007 [16-18]. 

The tariff price in shoulder surgery was felt to be "inaccurate," with the arthroscopic Healthcare Resource Group (HRG) being too general. It failed to address the extra time and expense of equipment utilized in some procedures [13]. The cost has been found to be extremely sensitive to operation length and delays; even teaching can cause a "profitable operation" to lose money for the trust [13].

Surgical targets are useful to ensure that procedures impacting patients’ quality-adjusted life years (QALYs) are delivered. From 2012, the operational target was that 92% of patients should be treated within 18 weeks, but this target was last achieved in September 2015 [19]. In December 2019, only 84% of patients were treated within 18 weeks, and this had fallen to 57% by December 2023 [19]. At the other extreme, some patients are waiting longer than 2 years. This number was reduced to 282 for all disciplines by December 2023; those remaining often involve extraordinarily complex cases treated in specialist centres with bespoke implants or equipment, so the capacity to treat them remains significantly limited [19]. Also in December 2023, there were 239,076 waits between 1 and 1.25 years and 337,450 incomplete pathways with waits of more than one year, reflecting the immense pressure that the NHS finds itself under to deliver the increasing burden of care, partly due to the aging demographic. In this regard, the department carried a significant loss for delivering fragility fracture care, which acts as a disincentive to providing that care, especially as in the same year the care delivered through the National Hip Fracture Database was commended for having one of the better adjusted mortality rates. There is a lack of investment in infrastructure to support care delivery, necessitating bargaining for the same service on the weekend as during the week, and being told that it is too expensive to provide a dedicated, all-day weekend trauma list.

The trust’s target for surgical waiting times in elective care has been reduced from 65 weeks to 54 weeks, placing significant pressure on services to deliver this care. This pressure is exacerbated by a reduced profit per case, as some procedures are being performed as inpatients rather than as day cases and on additional lists during unsocial hours by teams hired through locum agencies.

Orthopaedic services have become more efficient over time; for instance, the length of a hospital stay for primary hip and knee replacements has decreased from 15 days in 1997 to about 5.5 days in 2014 [20]. With recent advances and successful pilots, many surgeons and hospitals now undertake these surgeries as day cases [21]. Some elective shoulder replacements are also now delivered as day case surgery, which results in a shorter stay and reduced costs, much like day case trauma surgery [4,9,22].

Despite these efficiencies, there is a greater demand for trauma care, which is outstripping that provided even for elective care. The value of care has been expressed as patient-centred outcomes (safety and effectiveness; quality) divided by the related costs of care or per unit cost [23]. Predicting how long a case may take based on surgical complexity, the surgeon’s experience, the anaesthetist, and the scrub team remains difficult. Furthermore, there is an adverse cost pressure on training the team, all based on time.

A system that nurtures safety over speed might gauge daily case volume attained through complexity. This can be indexed against the volume of cases waiting (less tariff for more volume waiting) rather than time, which could lead to a poorly executed and rushed operation. A different paradigm is required to incentivize more productivity and help maintain recruitment and staff retention within theatres.

Service improvement models can help drive change towards a more financially robust model that delivers care for different patterns of injury more effectively within orthopaedic departments through audits and other quality improvement models [24]. The development of elective care units has also redistributed a considerable proportion of the profitable work through to elective care and private health providers [3]. The care model that a hospital adopts depends on the financial model that supports that care. Limited subspecialties can attract investment in services based on profitability, with trauma, foot and ankle, shoulder and elbow, and hand units struggling to get the same focus as elective hip and knee surgery. A change in the model may encourage a change in workflow so that there is an evening up in care for ambulatory trauma and elective care.

## Conclusions

Transitioning away from a time-based model towards an ambulatory pathway delivered care, which is more cost-effective, can improve the delivery of orthopaedic care. However, to aid this change, the tariffs associated with this care model must be reviewed in order to help finance a more efficient and profitable service that delivers high-quality care to the patients. At present, there does appear to be a financial model that supports ambulatory care for trauma other than in shoulder surgery, although wrist surgery showed a reduced loss within a trust but not within a region for ambulatory care. We recognise that this is a single-centre descriptive study and analysis without sensitivity analysis or adjustments for case mix. Therefore, further studies on this subject would help build up a robust platform for the proposed models.
